# Beyond target lesions: Prognostic value of longitudinal AI-derived whole-body [^1^⁸F]FDG PET/CT metrics in metastatic melanoma

**DOI:** 10.1007/s00259-026-07915-1

**Published:** 2026-05-26

**Authors:** Christos Sachpekidis, Theodoros P. Vagenas, Marilena Müller, Dimitrios Strauss, Annette Kopp-Schneider, Leyun Pan, Jessica C. Hassel, George K. Matsopoulos, Antonia Dimitrakopoulou-Strauss

**Affiliations:** 1 Clinical Cooperation Unit Nuclear Medicine, German Cancer Research Center - Deutsches Krebsforschungszentrum (DKFZ), Im Neuenheimer Feld 280, 69210 Heidelberg, Germany; 2 School of Electrical and Computer Engineering, National Technical University of Athens, Athens, Greece; 3 Department of Biostatistics, German Cancer Research Center - Deutsches Krebsforschungszentrum (DKFZ), Heidelberg, Germany; 4 Department of Diagnostic and Interventional Radiology, University Clinic Heidelberg, Heidelberg, Germany; 5 Medical Faculty Heidelberg, Department of Dermatology, Heidelberg University, Heidelberg, Germany; 6 National Center for Tumor Diseases (NCT), NCT Heidelberg, a partnership between, DKFZ and University Hospital Heidelberg, Heidelberg, Germany

**Keywords:** Metastatic melanoma, Immune checkpoint inhibitors (ICIs), Treatment response evaluation, Longitudinal analysis, [^18^F]FDG PET/CT, Image segmentation, Representation learning, Artificial intelligence

## Abstract

**Purpose:**

To investigate the prognostic value of an artificial intelligence (AI)–based semi-automated tool for longitudinal whole-body quantification of total metabolic tumor volume (TMTV) and total lesion glycolysis (TLG) on [^1^⁸F]FDG PET/CT in patients with metastatic melanoma undergoing immune checkpoint inhibitor (ICI) therapy, and to assess its prognostic relevance alongside established PET-based metabolic response criteria.

**Methods:**

Forty-three patients with unresectable metastatic melanoma treated with ICIs underwent [^1^⁸F]FDG PET/CT at baseline (n = 43), after two cycles (interim; n = 41), and after four cycles of therapy (late; n = 43). Whole-body tumor segmentation was performed using a previously validated AI-based framework combining ensemble unsupervised segmentation and deep representation learning, followed by expert review. TMTV and TLG were calculated for each time point. Overall survival (OS) was analyzed using Kaplan–Meier estimates, log-rank tests, and Cox proportional hazards regression. Multivariable models included LDH, AJCC stage, and ECOG performance status. Metabolic response was additionally assessed using EORTC, PERCIST, PERCIMT, imPERCIST5, and iPERCIST criteria.

**Results:**

Median follow up [95% CI] of the patient cohort from the date of baseline PET/CT was 97.8 months [90.1—134.5 months]. Automated whole-body segmentation and volumetric quantification were feasible in all examinations. In univariable Cox analysis, baseline TMTV and TLG showed non-significant associations with OS. At interim and late PET/CT, higher TMTV and TLG were significantly associated with worse OS (interim: p = 0.04 for both; late: p = 0.03 for TMTV, p = 0.02 for TLG). Median-based dichotomization confirmed shorter OS in patients with elevated volumetric parameters with a statistically significant association for TLG (logrank p = 0.021) and a trend toward significance for TMTV (logrank p = 0.10) at baseline. At interim imaging, both TMTV (logrank p = 0.01) and TLG (logrank p = 0.04) were significantly associated with worse OS, with the strongest associations observed at late follow-up (both logrank p = 0.009). In multivariable analysis, elevated late TMTV and TLG remained independently associated with poorer OS, alongside increased LDH, whereas lower ECOG performance status independently predicted improved survival. Among the applied metabolic response criteria, at both interim and late PET/CT, survival curves demonstrated a non-significant trend toward longer OS in responders (CMR + PMR) compared with non-responders (SMD + PMD), with this trend being most pronounced for PERCIMT.

**Conclusion:**

AI-based longitudinal whole-body quantification of TMTV and TLG on [^1^⁸F]FDG PET/CT appears to provide independent prognostic information in metastatic melanoma patients undergoing immunotherapy. In particular, volumetric PET metrics derived from late follow-up imaging demonstrate robust association with OS and may outperform conventional response criteria. AI-driven whole-body tumor burden assessment may enhance objective risk stratification and support treatment monitoring in the era of immunotherapy.

**Supplementary Information:**

The online version contains supplementary material available at 10.1007/s00259-026-07915-1.

## Introduction

Metastatic melanoma is a highly aggressive malignancy frequently characterized by extensive tumor burden and widespread dissemination, often involving multiple organ systems with numerous metastatic lesions. ^18^F-fluorodeoxyglucose ([^18^F]FDG) positron emission tomography/computed tomography (PET/CT) represents the imaging modality of choice for the detection, staging, and surveillance of metastatic disease in advanced melanoma, owing to its high sensitivity for metabolically active lesions [1, 2]. However, the typically multifocal disease pattern poses substantial challenges for accurate assessment of total tumor burden and disease extent, as well as for reliable imaging-based response evaluation. Conventional response assessment approaches, which rely on longitudinal evaluation of a limited number of target lesions, may therefore inadequately reflect true whole-body tumor burden in this setting [3]. Consequently, quantitative imaging biomarkers capable of capturing global disease extent rather than individual lesions are of particular interest [4].

The introduction of immune checkpoint inhibitors (ICIs) has led to a paradigm shift in oncology and has profoundly altered the therapeutic landscape of advanced melanoma [5]. Over the past decade, immunotherapies targeting immune checkpoint pathways have resulted in unprecedented response rates and survival benefits in patients with metastatic melanoma and are now considered standard of care [6–9]. Despite these advances, responses to immunotherapy are often heterogeneous and associated with atypical response patterns, including pseudoprogression, delayed responses, and mixed responses across different lesions, as well as the occurrence of immune-related adverse events (irAEs). These phenomena complicate treatment evaluation and may lead to misclassification of disease progression, particularly during early follow-up, when conventional response criteria are frequently insufficient [10–19].

In routine clinical practice, estimation of tumor extent and assessment of response to immunotherapy using [^18^F]FDG PET/CT remain largely visual and partly subjective in nature, with quantitative, thus more objective, analyses predominantly limited to the semi-quantitative standardized uptake value (SUV). Beyond visual interpretation, whole-body PET-derived parameters such as total metabolic tumor volume (TMTV) and total lesion glycolysis (TLG) provide integrated measures of global metabolic tumor burden and may better reflect the complex and heterogeneous response patterns observed during immunotherapy. Previous studies have demonstrated that baseline and longitudinal MTV and TLG are associated with progression-free survival (PFS) and overall survival (OS) in melanoma patients treated with ICIs [3, 20, 21]. Nevertheless, routine clinical implementation of these parameters remains limited by the labor-intensive and operator-dependent nature of lesion segmentation, particularly in patients with high tumor burden and multiple metastases, as well as the lack of clinically validated and widely available software solutions.

Recent advances in artificial intelligence (AI) have enabled the development of automated and semi-automated tools for lesion detection and segmentation on whole-body [^18^F]FDG PET/CT images, offering the potential to improve workflow efficiency, reduce inter-observer variability, and enhance the reproducibility of quantitative PET metrics [22, 23]. An AI-based decision support system for the analysis of whole-body FDG PET/CT in metastatic melanoma has previously been introduced and technically validated by our group, demonstrating robust performance in metastasis identification and disease representation through the incorporation of semantic and three-dimensional positional features [24, 25]. Importantly, this approach enables standardized and reproducible calculation of whole-body TMTV and TLG across serial PET/CT examinations [26]. While the diagnostic and technical performance of this AI-based tool has been proven, its clinical value as a prognostic instrument has not yet been investigated. In particular, it remains unclear whether AI-derived whole-body TMTV and TLG assessed at baseline and during follow-up can provide meaningful prognostic information in the setting of immunotherapy.

The aim of the present study was therefore to investigate, for the first time, the prognostic role of an AI-based semi-automated tool for longitudinal whole-body TMTV and TLG calculation on baseline and follow-up [^18^F]FDG PET/CT scans in patients with metastatic melanoma undergoing therapy with ICIs, and to compare its performance with different PET/CT-based metabolic response criteria.

## Materials and methods

### Patients

Forty-three patients (26 males, 17 females; mean age 59.1 ± 13.3 years) with metastatic melanoma undergoing immunotherapy with ICIs were enrolled in this retrospective analysis of a prospectively conducted study. A subset of these patients has been previously analyzed in other publications; however, those studies addressed different scientific questions and applied different methodological approaches compared with the present work [27, 28]. Patients received cytotoxic T-lymphocyte-associated antigen 4 (CTLA-4) inhibitors (ipilimumab), programmed cell death protein 1 (PD-1) inhibitors (pembrolizumab or nivolumab), or a combination of CTLA-4 and PD-1 inhibitors (nivolumab/ipilimumab). Ipilimumab was administered intravenously at a dose of 3 mg/kg every 3 weeks for a total of four doses. Pembrolizumab was administered intravenously at a dose of 2 mg/kg every 3 weeks. Nivolumab was administered intravenously at a dose of 3 mg/kg every 2 weeks. Combination immunotherapy consisted of an induction phase with four cycles of nivolumab (1 mg/kg) and ipilimumab (3 mg/kg) administered every 3 weeks, followed by single-agent nivolumab administration (3 mg/kg) every 2 weeks. Patients had not received chemotherapy for at least one month prior to the baseline PET/CT examination. None of the patients had a history of diabetes mellitus. All patients provided written informed consent for study participation and for the use of their medical data. The study protocol was approved by the Ethical Committee of the University of Heidelberg (S-107/2012) and by the Federal Agency for Radiation Protection (Bundesamt für Strahlenschutz, Z 5–22,463/2–2012–016).

### [^18^F]FDG PET/CT data acquisition

[^18^F]FDG PET/CT examinations were performed at three predefined time points: prior to initiation of immunotherapy (baseline PET/CT; n = 43), after two treatment cycles (interim PET/CT; 4–6 weeks after treatment initiation; n = 41), and after four treatment cycles (late PET/CT; 8–12 weeks after treatment initiation; n = 43).

Patients underwent whole body PET/CT imaging 60 min after intravenous administration of up to 250 MBq of [^18^F]FDG. Image acquisition was performed from the head to the feet with an acquisition time of 2 min per bed position. All scans were acquired using a dedicated PET/CT system (Biograph mCT S128, Siemens Healthineers, Erlangen, Germany) with an axial field of view of 21.6 cm, equipped with TruePoint and TrueV technology, and operated in three-dimensional mode.

A low-dose CT scan (120 kV, 30 effective mAs) was acquired for attenuation correction of PET data and for anatomical localisation. PET images were reconstructed using an ordered subset expectation maximization (OSEM) algorithm with point spread function modeling (2 iterations, 21 subsets) and time-of-flight (TOF) information. All PET images were attenuation-corrected, and an image matrix of 400 × 400 pixels was applied.

### [^18^F]FDG PET/CT data analysis

#### Automated PET/CT data analysis and quantification

In this study, we validate an automatic segmentation method for delineating true tumor lesions in whole-body [^18^F]FDG-PET/CT images and evaluate its prognostic value for survival outcomes. Tumor segmentation is performed using a previously developed two-stage framework [24, 25]. First, an unsupervised ensemble-based segmentation algorithm is applied to identify regions of increased [^18^F]FDG uptake on PET/CT [24]. Subsequently, these candidate regions are classified as tumor lesions or non-tumor findings, using a representation-learning model trained to extract discriminative deep features [25]. In this study, segmentation masks generated by the automated pipeline were reviewed and corrected by an experienced nuclear medicine physician (C.S.), after which the corresponding quantitative metrics were calculated.

Initially, an unsupervised segmentation of high-uptake regions is obtained by ensembling three complementary submodules: (i) a clustering-based segmentation approach, (ii) a custom CT-guided region-growing method, and (iii) a PET-specific region-growing method. The output mask is generated by applying the S.T.A.P.L.E. method to fuse these three outputs into a single consensus segmentation [29]. The second step aims to extract highly informative feature representations, combining semantic features with position encodings, capable of distinguishing true tumor lesions from physiological uptake. The complete pipeline ultimately produces a whole-body [^1^⁸F]FDG PET/CT mask delineating true tumor lesions.

The representation learning framework generates embeddings that capture tumor-related characteristics using a deep learning scheme. Specifically, a backbone network, ResNet18, is trained under the VICReg scheme to learn these representations. The main building blocks of the framework are presented below:

- Augmentation and Sampling module: It generates paired ROIs (including augmented views) to learn class-informative embeddings, while applying strategies to mitigate class imbalance and reduce inter-class similarity.
- Position Encoding Block (PEB): For each ROI, a positional vector encoding the centroid, size, and rotation is computed and fed into the PEB to inject spatial information into the semantic representation. The resulting fused vector is the final representation for the ROI.
- VICReg-based learning scheme [30]: This learning scheme enables the extraction of informative representations through the invariance, variance, and covariance components of its loss function.

Finally, the trained representation scheme is employed to classify extracted ROIs. The representations extracted from each region were subsequently classified as tumor or non-tumor using a trained multilayer perceptron (MLP) classifier.

Semiautomatic corrections: Using these labels, an automated mask containing only tumor lesions is produced. An experienced nuclear medicine physician (C.S.) applied manual corrections to this mask to obtain the final tumor mask for the whole-body metrics calculation, namely total MTV (TMTV) and TLG. Prior to classification, large organs, including the brain, kidneys and bladder, are automatically excluded using CT-derived organ masks generated with TotalSegmentator [31]. Manual adjustments include removal of false-positive regions, such as ureters and bowel uptake, addition of missed ROIs, and reduction of spillover effects from adjacent structures due to the limited spatial resolution of PET images.

### Application of metabolic response criteria

[^18^F]FDG PET/CT images were reviewed and interpreted in consensus by two experienced nuclear medicine physicians with expertise in melanoma imaging (C.S., A.D.S.), as previously described [27]. Assessment of metabolic response to immunotherapy was performed according to the following established PET/CT–based response criteria:

- European Organization for Research and Treatment of Cancer (EORTC) 1999 criteria [32]
- Positron Emission Tomography Response Criteria in Solid Tumors (PERCIST) [33]
- PET Response Evaluation Criteria for IMmunoTherapy (PERCIMT) [34]
- Immunotherapy-modified PERCIST (imPERCIST5) [35]
- Immune PERCIST (iPERCIST) [36]

Based on these criteria, metabolic response to immunotherapy was categorized as complete metabolic response (CMR), partial metabolic response (PMR), stable metabolic disease (SMD), or progressive metabolic disease (PMD). In addition, a composite outcome measure was defined as response rate (RR), comparing responders (CMR + PMR) versus non-responders (SMD + PMD).

### Statistical analysis

OS was defined as the time from each PET/CT (baseline, interim, late) to death from any cause or last follow-up. For quantitative analyses TMTV and TLG measurements were log-transformed as their distributions were found to be heavily skewed. Measurements equal to zero were set to an offset of 0.01 for TMTV and 0.1 for TLG. Kaplan–Meier estimates were generated and median survival times were estimated. Median follow-up time was estimated by inverse Kaplan–Meier method. For univariable comparison of OS, log-rank test was used, dichotomizing the quantitative variables at their median. Univariable Cox proportional hazards regression analysis was applied for the different log-transformed TMTV and TLG measurements. Multivariable Cox proportional hazards regression analysis was applied to investigate association between survival time of patients and multiple predictors simultaneously. A time-dependent Cox model was used to assess the longitudinal association of TMTV and TLG with OS. In addition, several Cox proportional hazards regression analyses were performed to evaluate longitudinal effects, including changes in marker values between baseline and interim assessments, as well as between baseline and late follow-up. For parameters highly correlated with each other, such as TMTV and TLG, only one was included at a time in the model. Statistical analyses were performed using R software (version 4.0.3; packages: *survival*, *ggsurvfit*, and *prodlim*). Due to the moderate cohort size, no correction for multiple testing could be applied, even though multiple comparisons were performed across imaging time points and PET-derived parameters. A two-sided p-value < 0.05 was considered statistically significant, but should only be interpreted as exploratory finding.

## Results

### Patient cohort

Baseline patient characteristics are summarized in Table 1. According to the American Joint Committee on Cancer (AJCC) staging system, 1 patient (2%) was classified as M0 (stage III), 7 patients (16%) as M1a, 8 patients (19%) as M1b, 26 patients (60%) as M1c, and 1 patient (2%) as M1d. Twenty-five patients (58.1%) received first-line systemic treatment with ICIs, whereas 18 patients (41.9%) had received at least one prior line of systemic treatment. Fourteen patients (32.6%) had previously undergone radiotherapy. Regarding immunotherapy regimens, 25 patients received ipilimumab, 5 received pembrolizumab, 2 received nivolumab, and 11 patients were treated with a combination of nivolumab and ipilimumab. The mean baseline lactate dehydrogenase (LDH) level was 256.3 U/l. Median follow up [95% CI] of the patient cohort from the date of baseline PET/CT was 97.8 months [90.1—134.5 months]. At the time of data cutoff, the median OS was 38.4 months [17.5—NA] and 28 patients (65%) had died.

**Table 1:** Baseline patient characteristics

Patient characteristics	Value
n	43
Sex, n (%)	
Male	26 (60.5%)
Female	17 (39.5%)
Median age, years (IQR)	58.4 (51.9–67.8)
Median LDH, U/l (IQR)	223.0 (194.5–272.0)
Median S100, μg/l (IQR)	0.077 (0.048–0.131)
Previous systemic therapy, n (%)	
Yes	18 (41.9)
No	25 (58.1)
Previous chemotherapy, n (%)	
Yes	12 (27.9)
No	31 (72.1)
Previous radiotherapy, n (%)	
Yes	14 (32.6)
No	29 (67.4)
M stage, n (%)	
M0 (Stage III)	1 (2.3%)
M1a	7 (16.3%)
M1b	8 (18.6%)
M1c	26 (60.5%)
M1d	1 (2.3%)
ECOG performance status, n (%)	
0	35 (81.4)
1	7 (16.3)
2	1 (2.3)
ICI treatment, n (%)	
Ipilimumab	25 (58.1)
Ipilimumab/Nivolumab	11 (25.6)
Nivolumab	2 (4.7)
Pembrolizumab	5 (11.6)

*LDH,* lactate dehydrogenase; *IQR,* interquartile range; *ICI,* immune checkpoint inhibitor; *TMTV,* total metabolic tumor volume; *TLG,* total lesion glycolysis

### Automated [^18^F]FDG PET/CT quantification

**Fig. 1:**
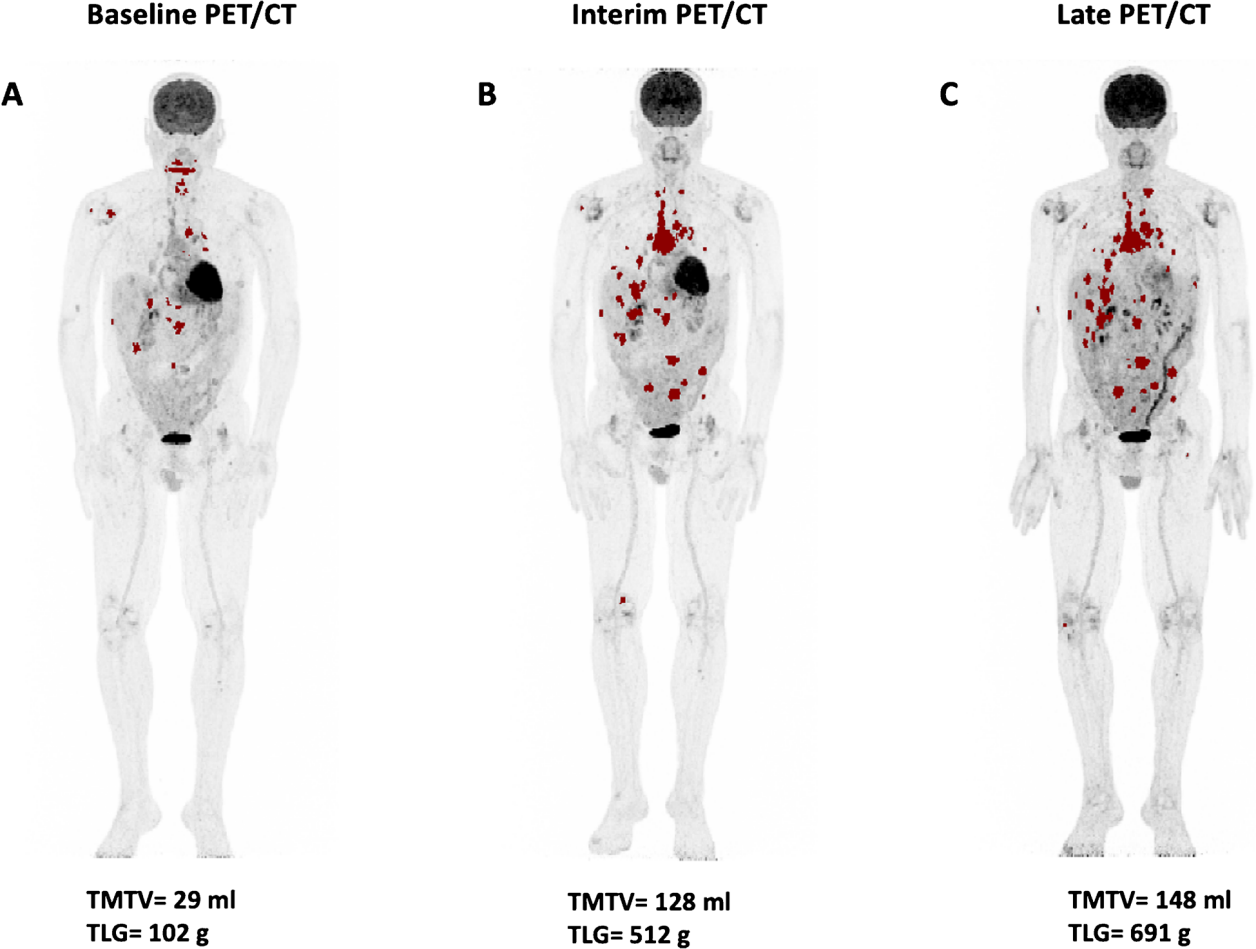
Representative example of AI-driven automated calculation of whole-body TMTV and TLG in a metastatic melanoma patient at baseline (A), after two cycles (B), and after four cycles (C) of ipilimumab treatment. Both metabolic parameters showed marked increases from baseline PET/CT (TMTV = 29 mL, TLG = 102 g; A) to interim follow-up PET/CT (TMTV = 128 mL, TLG = 512 g; B), and to late follow-up PET/CT (TMTV = 148 mL, TLG = 691 g; C). The patient demonstrated PMD both on interim and late follow-up PET/CT based on all applied metabolic criteria

**Fig. 2:**
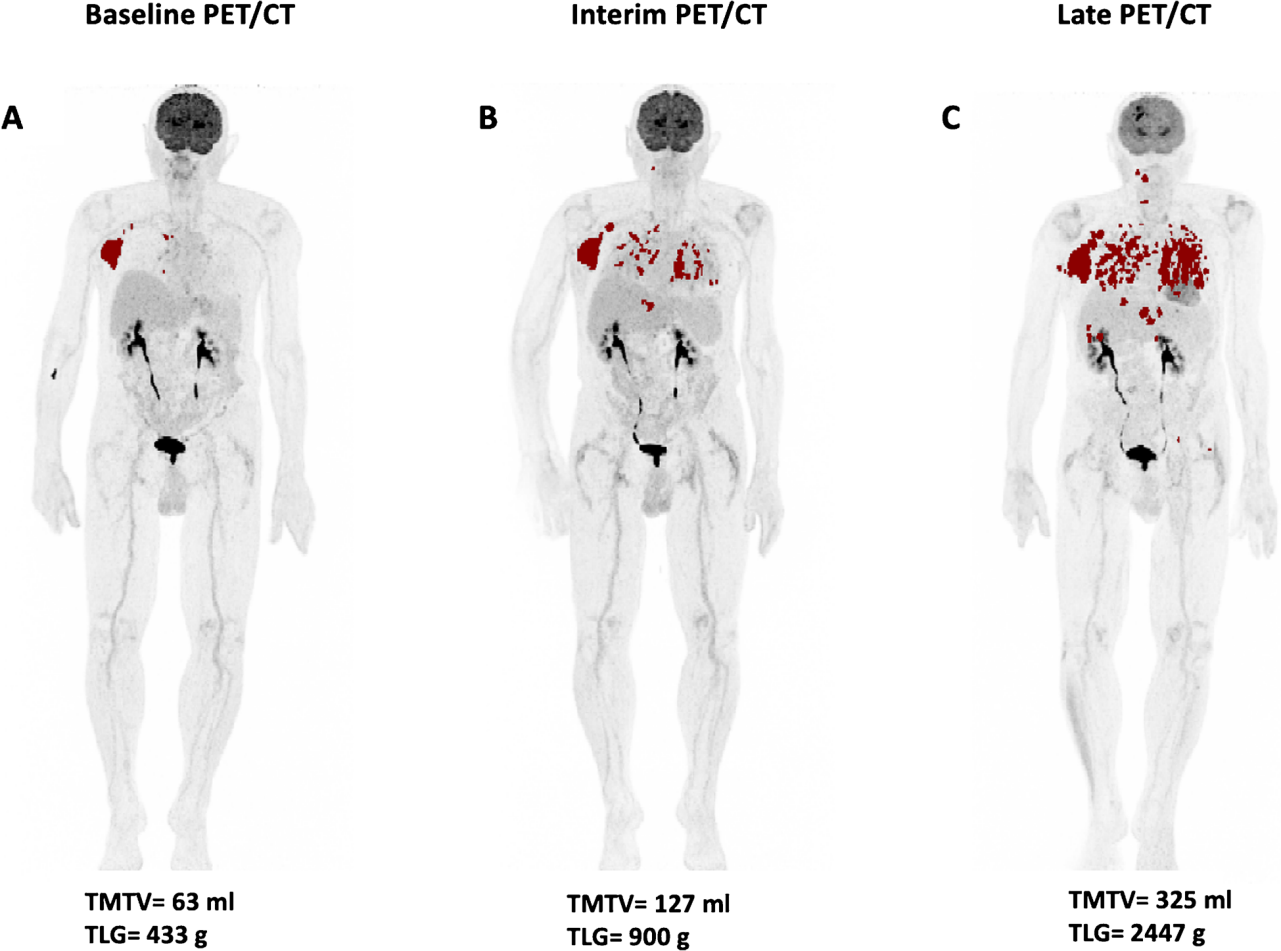
Representative example of AI-driven automated calculation of whole-body TMTV and TLG in a metastatic melanoma patient at baseline (A), after two cycles (B), and after four cycles (C) of pembrolizumab treatment. Both metabolic parameters showed markedincreases from baseline PET/CT (TMTV = 63 mL, TLG = 433 g; A) to interim follow-up PET/CT (TMTV = 127 mL, TLG = 900 g; B), and to late follow-up PET/CT (TMTV = 325 mL, TLG = 2447 g; C). The patient demonstrated PMD both on interim and late follow-up PET/CT based on all applied metabolic criteria

Whole-body segmentation and automated volumetric quantification of TMTV and TLG were successfully performed in all PET/CT studies. Non-significant changes were observed for the PET parameters in serial imaging. The corresponding results are summarized in Table 2. Representative examples illustrating the application of the AI tool in two patients from the study cohort are shown in Figs. 1 and 2.

**Table 2:** Longitudinal descriptive statistics of total metabolic tumor volume (TMTV) and total lesion glycolysis (TLG) derived from AI-based analysis at baseline, interim, and late PET/CT

Parameter	Baseline PET/CT			Interim PET/CT				Late PET/CT	
	*Mean (SD)*		*Median*	*Mean (SD)*			*Median*	*Mean (SD)*	*Median*
TMTV (ml)	59 (108)	9		76 (155)	10			135 (319)	8
TLG (g)	461 (855)	55		584 (1368)		49		908 (2138)	48

*TMTV,* total metabolic tumor volume; *TLG,* total lesion glycolysis

Univariable Cox proportional hazards regression analysis for OS was conducted including TMTV and TLG (Table 3). On baseline PET/CT, both parameters demonstrated an adverse, though non-significant, association with OS (p = 0.13 for TMTV; p = 0.18 for TLG). On interim PET/CT, both parameters were significantly associated with worse OS (p = 0.04 for both). Similarly, on late PET/CT, both TMTV and TLG showed a significant adverse association with OS (p = 0.03 for TMTV; p = 0.02 for TLG).

**Table 3:** Prognostic significance of longitudinal AI-derived PET biomarkers (TMTV and TLG) for overall survival in univariable analysis

		**Overall survival**	
	**PET parameters**	**HR (95% CI)**	***p-value***
*Baseline PET*	TMTV (mL)	1.1260 (0.9673–1.3110)	0.1260
	TLG (g)	1.1025 (0.9567–1.2700)	0.1780
*Interim PET*	TMTV (mL)	1.1433 (1.0090–1.2950)	0.0354*
	TLG (g)	1.1316 (1.0030–1.2760)	0.0443*
*Late PET*	TMTV (mL)	1.1303 (1.0130–1.2610)	0.0280*
	TLG(g)	1.1328 (1.0170–1.2620)	0.0240*

*HR,* hazard ratio; *95% CI,* 95% confidence intervals; *TMTV,* total metabolic tumor volume; *TLG,* total lesion glycolysis

^*^Statistically significant correlation

**Fig. 3:**
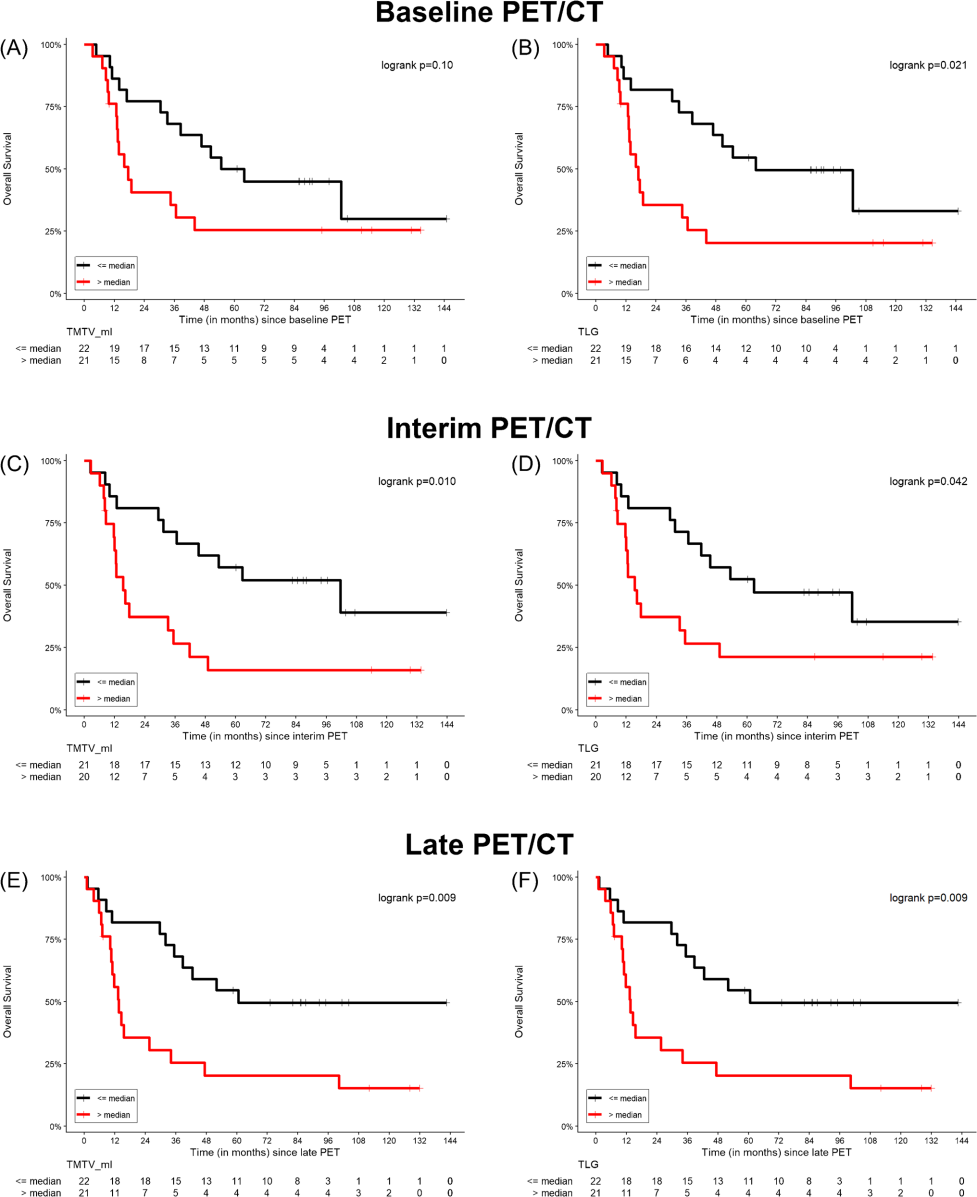
Kaplan–Meier estimates of OS stratified by longitudinal AI-derived PET biomarkers (TMTV and TLG) assessed at baseline (A–B), interim (C–D), and late PET/CT (E–F). The numbers of patients at risk for each group and corresponding time points are displayed below the plots

To further evaluate their prognostic impact, automated PET parameters were dichotomized at the median and analyzed using the Kaplan–Meier method with log-rank testing (Table 4). At baseline PET/CT, Kaplan–Meier analysis demonstrated a non-significant trend for TMTV (p = 0.10) and a significant association for TLG (p = 0.021) (Fig. 3A-B). On interim PET/CT, patients with TMTV and TLG values above the median exhibited significantly shorter OS compared with those below the median (p = 0.01 for TMTV; p = 0.04 for TLG) (Fig. 3C-D). Likewise, on late PET/CT, elevated TMTV and TLG were both significantly associated with reduced OS (p = 0.009 for both) (Fig. 3E-F).

**Table 4:** Results of survival analysis based on longitudinal AI-derived PET biomarkers (TMTV and TLG) using the Kaplan–Meier method with log-rank testing

**Parameter**		**Baseline PET/CT**		**Interim PET/CT**		**Late PET/CT**	
		**Median OS [95% CI]**	***p value***	**Median OS [95% CI]**	***p value***	**Median OS [95% CI]**	***p value***
**TMTV**	≤ median	59.3 months [38.4—NA]	0.10	101.5 months [36.6—NA]	0.01*	60.7 months [38.7—NA]	0.009*
	> median	17.5 months [13.1—NA]		15.4 months [11.9–48.9]		13.5 months [10.6–47.4]	
**TLG**	≤ median	63.9 months [46.8—NA]	0.021*	62.7 months [36.6—NA]	0.042*	60.7 months [38.7—NA]	0.009*
	> median	17.0 months [13.1–44.0]		15.4 months [11.9—NA]		13.5 months [10.6–47.4]	

*TMTV,* total metabolic tumor volume; *TLG,* total lesion glycolysis; *OS,* overall survival

^*^ Statistically significant difference

In multivariable analysis, the impact of longitudinal AI-derived PET parameters and baseline clinical variables—including AJCC stage, ECOG performance status, and LDH -was assessed simultaneously. High TMTV and TLG values on late PET/CT remained independently associated with poorer survival. Increased LDH levels were likewise independently associated with worse OS, whereas lower ECOG performance status was independently associated with improved OS (Table 5).

**Table 5:** Multivariate Cox regression analysis of clinical parameters and AI-derived PET metrics. Given the strong correlation between TMTV and TLG, two separate models were fitted, each incorporating only one PET parameter (Model 1: TMTV; Model 2: TLG)

Parameters	Model 1 (incl. PET TMTV)			Model 2 (incl. PET TLG)		
		**Overall survival**				
	**HR (95% CI)**	***p-value***		**HR (95% CI)**		***p-value***
*Baseline PET*						
AJCC						
High AJCC	1.032 (0.464–2.292)		0.9391	1.039 (0.464–2.329)		0.9259
ECOG score						
Low ECOG score	0.169 (0.064–0.448)		0.0003*	0.165 (0.062–0.436)		0.0003*
LDH	1.004 (1.000–1.007)		0.0287*	1.004 (1.000–1.007)		0.0265*
PET parameter (TMTV/TLG)	1.047 (0.897–1.222)		0.5598	1.032 (0.891–1.195)		0.6720
*Interim PET*						
AJCC						
High AJCC	1.018 (0.436–2.377)		0.9680	1.032 (0.433–2.460)		0.9441
ECOG score						
Low ECOG score	0.196 (0.072–0.533)		0.0014*	0.191 (0.070–0.520)		0.0012*
LDH	1.003 (1.000–1.007)		0.0524	1.003 (1.000–1.007)		0.0550
PET parameter (TMTV/TLG)	1.088 (0.953–1.243)		0.2112	1.079 (0.944–1.233)		0.2648
*Late PET*						
AJCC						
High AJCC	1.199 (0.530–2.713)		0.6639	1.263 (0.552–2.890)		0.5805
ECOG score						
Low ECOG score	0.162 (0.063–0.420)		0.0002*	0.170 (0.066–0.438)		0.0002*
LDH	1.003 (0.999–1.006)		0.0996	1.003 (0.999–1.006)		0.0971
PET parameter (TMTV/TLG)	1.121 (1.001–1.255)		0.0474*	1.121 (1.001–1.255)		0.0481*

^*^Statistically significant correlation

*TMTV,* metabolic tumor volume; *TLG,* total lesion glycolysis; *HR,* hazard ratio; *95% CI,* 95% confidence intervals; *AJCC,* American Joint Committee on Cancer; *ECOG,* Eastern Cooperative Oncology Group; *LDH,* lactate dehydrogenase

High AJCC is defined by M1c and M1d patients (vs. M0, M1a and M1b)

Low ECOG score is defined by score 0 (vs. score 1 and score 2)

Neither the longitudinal change in TMTV (p = 0.16) nor that in TLG (p = 0.17) had a significant impact on OS, either in the time-dependent Cox model or in the Cox proportional hazards model assessing differences between time points. This lack of significance may be related to the moderate sample size.

### Application of metabolic response criteria

Metabolic response to immunotherapy was evaluated on interim and late PET/CT according to established PET/CT-based response criteria (Table 6). A composite response rate (RR) was defined, and its association with OS was assessed using Kaplan–Meier survival analysis (Suppl. Tables 1–2).

**Table 6:** Interim and late PET/CT response assessment to immunotherapy according to the applied metabolic criteria. Values refer to number of patients (%)

	**EORTC**	**PERCIST**	**PERCIMT**	**imPERCIST5**	**iPERCIST**
*Interim PET/CT*					
CMR	1 (2.3%)	3 (7.0%)	1 (2.3%)	3 (7.0%)	3 (7.0%)
PMR	9 (20.9%)	6 (14.0%)	7 (16.3%)	7 (16.3%)	6 (14.0%)
SMD	9 (20.9%)	19 (44.2%)	25 (58.1%)	20 (46.5%)	19 (44.2%)
PMD	24 (55.8%)	15 (34.9%)	10 (23.3%)	13 (30.2%)	15 (34.9%)
*Late PET/CT*					
CMR	4 (9.3%)	5 (11.6%)	4 (9.3%)	4 (9.3%)	5 (11.6%)
PMR	9 (20.9%)	5 (11.6%)	10 (23.3%)	7 (16.3%)	5 (11.6%)
SMD	8 (18.6%)	10 (23.3%)	16 (37.2%)	13 (30.2%)	10 (23.3%)
PMD	22 (51.2%)	23 (53.5%)	13 (30.2%)	19 (44.2%)	23 (53.5%)

*EORTC,* European Organisation for Research and Treatment of Cancer criteria; *PERCIST,* PET Response Criteria in Solid Tumors; *PERCIMT,* PET Response Criteria in Melanoma; *imPERCIST5,* immunotherapy-modified PERCIST; *iPERCIST,* immunotherapy-modified PERCIST; *CMR,* complete metabolic response; *PMR,* partial metabolic response; *SMD,* stable metabolic disease; *PMD,* progressive metabolic disease

**Fig. 4:**
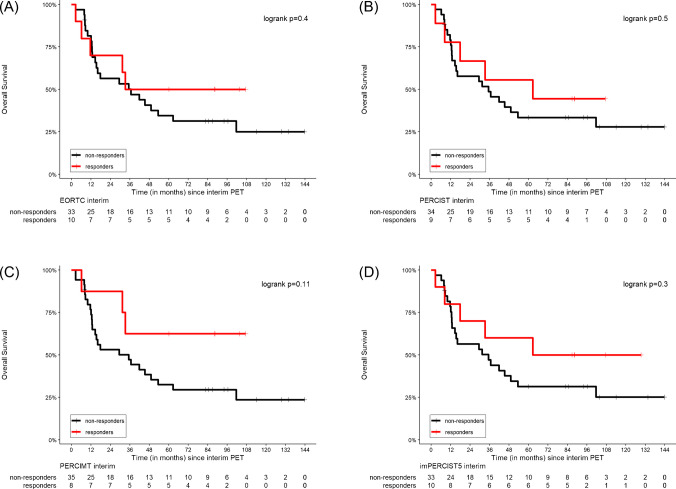
Kaplan–Meier analysis of OS based on interim PET/CT response classification according to different metabolic criteria. Survival curves stratified by response rate (RR) dichotomization using EORTC (A), PERCIST (B), PERCIMT (C), and imPERCIST5 (D). The numbers of patients at risk in each group and for the respective time-points are shown below the plots. iPERCIST curves are not presented, as survival results were identical to PERCIST, differing only in terminology (uPMD vs. PMD)

**Fig. 5:**
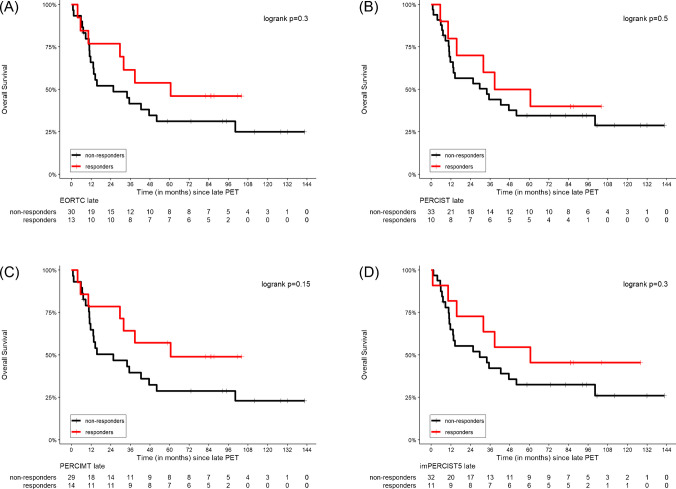
Kaplan–Meier analysis of OS based on late PET/CT response classification according to different metabolic criteria. Survival curves stratified by response rate (RR) dichotomization using EORTC (A), PERCIST (B), PERCIMT (C), and imPERCIST5 (D). The numbers of patients at risk in each group and for the respective time-points are shown below the plots. iPERCIST curves are not presented, as survival results were identical to PERCIST, differing only in terminology (cPMD vs. PMD)

At both interim and late PET/CT, survival curves across the applied criteria demonstrated a non-significant trend toward longer OS in responders (CMR + PMR) compared with non-responders (SMD + PMD). This trend was most pronounced for PERCIMT (Figs. 4 and 5).

## Discussion

In the present study, we investigated the prognostic significance of longitudinal, AI-derived whole-body metabolic tumor burden metrics in patients with metastatic melanoma undergoing immunotherapy with ICIs. The main findings are threefold. First, quantification of TMTV and TLG was technically feasible across serial [^18^F]FDG PET/CT examinations supported by a predominantly automated segmentation method. Second, univariable analysis demonstrated that high TMTV and TLG during treatment were significantly associated with poorer outcomes, a finding corroborated by Kaplan–Meier survival analysis. Third, in multivariable models adjusting for AJCC stage, ECOG performance status, and LDH, elevated late (after 4 cycles of ICIs) TMTV and TLG remained independently associated with reduced overall survival.

The advent of ICIs has transformed the therapeutic landscape for metastatic melanoma, but it has also introduced challenges for imaging-based treatment evaluation. Atypical response patterns—including pseudoprogression, delayed and mixed responses—limit the reliability of conventional response criteria that focus on a limited number of target lesions. Moreover, the typically multifocal, or even disseminated, disease distribution in metastatic melanoma makes precise tumor delineation labor-intensive and time-consuming, complicating accurate assessment of total tumor burden and reliable imaging-based response evaluation [3, 18]. In this context, global measures of metabolic tumor burden may better capture the complex biological behavior of melanoma under immunotherapy. Our results support this concept: whole-body volumetric PET metrics showed strong prognostic associations when assessed longitudinally, particularly at later treatment time points. This may be biologically explained by the observation that atypical response patterns on PET/CT—most notably pseudoprogression—tend to diminish over time [27]. Together, these findings highlight the potential of AI-derived volumetric PET parameters to complement conventional response evaluation and improve risk stratification in clinical practice.

Baseline TMTV and TLG demonstrated non-significant adverse trends for OS, whereas interim and late PET/CT parameters showed significant associations with survival. This temporal pattern suggests that dynamic changes in metabolic tumor burden under treatment may carry greater prognostic relevance than baseline tumor extent alone. It is conceivable that early metabolic adaptations to immunotherapy may reflect both tumor-intrinsic biology and host immune competence. This remains notable despite the well-known challenges in assessing ICI responses—including atypical response patterns and irAEs—partly due to the non-specific uptake of [^1^⁸F]FDG. Importantly, the independent association of late PET-derived TMTV and TLG with OS in multivariable models underscores their potential as robust imaging biomarkers in this clinical context, especially at later stages of ICI treatment.

Previous studies have explored AI-driven [^1^⁸F]FDG PET analysis for treatment response prediction and survival prognostication in melanoma patients undergoing immunotherapy. Basler et al. developed serial PET/CT-based radiomic models to enable early differentiation of pseudoprogression from true progression, achieving an area under the curve (AUC) of 0.82 using a multimodal model combining radiomic features with LDH and S100 (median follow-up 22 months) [37]. Dirks et al. introduced a fully automated deep learning algorithm for lesion detection and segmentation, demonstrating significant prognostic value of TMTV for PFS and OS, particularly in combination with clinical parameters [38, 39]. Similarly, Flaus et al. reported high predictive performance using machine-learning models based on pretreatment PET-derived radiomic features, achieving cross-validated AUCs of 0.87 for OS and 0.90 for PFS at a median follow-up 22.1 months [40].

While these AI-based approaches show substantial promise, several barriers may limit routine clinical implementation, particularly the reliance on segmentation methods sensitive to uptake variability and standardization issues. In contrast, we employed a previously validated AI segmentation tool combining clustering with customized region-growing techniques to extract high-uptake regions. Unlike radiomics-based approaches, which may require lesion-size thresholds and are sensitive to acquisition variability, our framework integrates semantic and positional deep feature representations, enabling more robust lesion characterization across heterogeneous disease patterns. The computational efficiency of the tool facilitated longitudinal whole-body quantification of TMTV and TLG and allowed inclusion of all detected lesions, extending prior approaches that focused on selected targets [41].

An important strength of our approach is the use of a standardized, previously validated AI-driven pipeline for lesion detection and segmentation. In earlier work [24, 25], the model was specifically designed to address challenges inherent to [^1^⁸F]FDG PET/CT and metastatic melanoma, including high tumor heterogeneity, limited annotated datasets, and false positives due to physiologic tracer uptake. The two-step framework integrating advanced image processing, machine learning, and state-of-the-art deep learning consistently demonstrated strong classification performance while reducing manual correction workload. In the present study, although minor manual adjustments were performed to ensure clinical plausibility, the high feasibility rate and consistent performance across serial examinations underline the robustness of the approach. Moreover, the exceptionally long follow-up period of our cohort strengthens the reliability of survival analyses.

In multivariable analysis, elevated LDH and impaired ECOG performance status retained their established prognostic relevance, while late TMTV and TLG provided independent and additive information. This finding is in line with previously mentioned studies [37, 39] and underscores that AI-derived imaging biomarkers should not be viewed in isolation but rather as complementary components of a multimodal prognostic framework integrating imaging, laboratory, and clinical data.

When evaluating AI-derived whole-body volumetric parameters alongside established PET-based metabolic response criteria—including conventional EORTC and PERCIST, as well as the immunotherapy-adapted PERCIMT, imPERCIST5, and iPERCIST frameworks— survival curves demonstrated a non-significant trend toward longer OS in responders (CMR + PMR) compared with non-responders (SMD + PMD), with this trend being most pronounced for PERCIMT. Notably, differences among these criteria relate not only to the definition of response patterns under immunotherapy, but also to the selection and definition of target lesions already at baseline imaging. These findings underscore the persistent challenge of accurately assessing treatment response under immunotherapy. While immunotherapy-adapted criteria attempt to account for immune-related response patterns, they remain categorical by design. In contrast, continuous volumetric parameters such as TMTV and TLG provide a more granular and objective quantification of global tumor burden, potentially capturing subtle yet clinically meaningful changes that may be overlooked by predefined response categories and by approaches focusing on metabolic changes in a limited number of target lesions or the appearance of a single new lesion as a criterion for metabolic progression.

Several limitations of this study should be acknowledged. First, this is a retrospective analysis of prospectively acquired data with a relatively limited sample size, restricting statistical power and extensive subgroup analyses. In addition, the prognostic analyses involved multiple PET-derived parameters assessed at several imaging time points, which further supports interpreting the findings with caution. Accordingly, the present results should be considered exploratory and hypothesis-generating until confirmed in larger, prospective, and independent cohorts. Nevertheless, the investigated cohort was relatively clinically homogeneous, imaged at standardized time points, and followed for an extended period, thereby ensuring methodological consistency and robust outcome assessment. Second, although automated segmentation improves reproducibility and reduces observer dependency, validation across institutions, scanner types, and acquisition protocols is necessary to establish generalizability. As emphasized in contemporary AI-based PET research, insufficient harmonization remains a major barrier to broader clinical implementation. Third, while previous studies from our group [24, 25] evaluated higher-order radiomic and deep-learning features, the present analysis deliberately focused on volumetric parameters to maintain clinical interpretability and specifically assess longitudinal performance of the AI tool. The integration of advanced radiomic features may provide additional prognostic value and warrants future investigation. Finally, most PET/CT findings were not histopathologically confirmed, reflecting real-world clinical practice. However, the long follow-up period enabled indirect validation through survival outcomes.

## Conclusion

Longitudinal AI-derived TMTV and TLG are feasible and clinically meaningful biomarkers in metastatic melanoma patients undergoing therapy with ICIs. Elevated values during immunotherapy independently predict poorer OS, providing incremental prognostic value beyond established clinical parameters. These findings support the growing role of AI-assisted PET quantification in precision oncology and highlight the importance of longitudinal metabolic tumor burden assessment in the immunotherapy setting. Larger prospective studies and external validation cohorts are needed to confirm the robustness and generalizability of these results.

## Supplementary Information

Below is the link to the electronic supplementary material.

Supplementary file1 (DOCX 24 KB)

## Data Availability

The datasets generated during and/or analysed during the current study are available from the corresponding author on reasonable request.
