# Supplementary material for: Beyond target lesions: Prognostic value of longitudinal AI-derived whole-body [1⁸F]FDG PET/CT metrics in metastatic melanoma
Source: Eur J Nucl Med Mol Imaging. 2026 May 26;53(10):5907–20. doi: 10.1007/s00259-026-07915-1 (PMC13421226; doi:10.1007/s00259-026-07915-1)
Supplement: Supplementary file 1 — Supplementary file1 (DOCX 24 KB) [file 259_2026_7915_MOESM1_ESM.docx]

**Suppl. Table 1** Results of survival analysis based on the applied metabolic criteria at interim PET/CT using the Kaplan–Meier method with log-rank testing. Values are presented as number of patients (%) and median overall survival (OS) with 95% confidence intervals (CI).

|  | **EORTC** | | **PERCIST** | | **PERCIMT** | | **imPERCIST5** | | **iPERCIST** | |
| --- | --- | --- | --- | --- | --- | --- | --- | --- | --- | --- |
|  | **No. patients** | **OS (months)** | **No. patients** | **OS**  **(months)** | **No. patients** | **OS**  **(months)** | **No. patients** | **OS**  **(months)** | **No. patients** | **OS**  **(months)** |
| **Metabolic response rate (RR)** | | | | | | | | | | |
| Responders (CMR, PMR) | 10 (23.3%) | 33.2 [11.6 - NA] | 9 (21.0%) | 62.7 [17.8 - NA] | 8 (18.6%) | NA [33.2 - NA] | 10 (23.3%) | 62.7 [17.8 - NA] | 9 (21.0%) | 62.7 [17.8 - NA] |
| Non-responders (SMD, PMD) | 33 (76.7%) | 36.6 [15.4 – 101.5] | 34 (79.0%) | 35.3 [14.5 - NA] | 35 (81.4%) | 35.3 [14.5 – 62.7] | 33 (76.7%) | 35.3 [14.5 – 101.5] | 34 (79.0%) | 35.3 [14.5 - NA] |
|  | *p=0.4* | | *p=0.5* | | *p=0.11* | | *p=0.3* | | *p=0.5* | |

*

The PMD results for iPERCIST refer to uPMD.

**Suppl. Table 2** Results of survival analysis based on the applied metabolic criteria at late PET/CT using the Kaplan–Meier method with log-rank testing. Values are presented as number of patients (%) and median overall survival (OS) with 95% confidence intervals (CI).

|  | **EORTC** | | **PERCIST** | | **PERCIMT** | | **imPERCIST5** | | **iPERCIST** | |
| --- | --- | --- | --- | --- | --- | --- | --- | --- | --- | --- |
|  | **No. patients** | **OS (months)** | **No. patients** | **OS**  **(months)** | **No. patients** | **OS**  **(months)** | **No. patients** | **OS**  **(months)** | **No. patients** | **OS**  **(months)** |
| **Metabolic response rate (RR)** | | | | | | | | | | |
| Responders (CMR, PMR) | 13 (30.2%) | 60.7 [29.6 - NA] | 10 (23.3%) | 49.7 [15.6 - NA] | 14 (32.6%) | 60.7 [31.9 - NA] | 11 (25.6%) | 60.7 [31.9 - NA] | 10 (23.3%) | 49.7 [15.6 - NA] |
| Non-responders (SMD, PMD) | 30 (69.8%) | 25.6 [13.2 – NA] | 33 (76.7%) | 34.0 [13.2 – NA] | 29 (67.4%) | 25.6 [11.7 – 100.2] | 32 (74.4%) | 29.6 [13.2 – NA] | 33 (76.7%) | 34.0 [13.2 – NA] |
|  | *p=0.3* | | *p=0.5* | | *p=0.15* | | *p=0.3* | | *p=0.5* | |

T

The PMD results for iPERCIST refer to cPMD.
